# Dr. James Clark Earle

**Published:** 1917-10

**Authors:** 


**Dr. James Clark Earle,** Buffalo 1887, died at his home in Olean, Aug. 7, aged 51. He was formerly Chief Surgeon of the Northern Division of the Pennsylvania R. R. and a member of the Olean City Council.

